# Endothelial Hey2 deletion reduces endothelial-to-mesenchymal transition and mitigates radiation proctitis in mice

**DOI:** 10.1038/s41598-017-05389-8

**Published:** 2017-07-10

**Authors:** Elodie Mintet, Jérémy Lavigne, Vincent Paget, Georges Tarlet, Valérie Buard, Olivier Guipaud, Jean-Christophe Sabourin, Maria-Luisa Iruela-Arispe, Fabien Milliat, Agnès François

**Affiliations:** 1 Institut de Radioprotection et de Sûreté Nucléaire (IRSN), Department of Radiobiology and Epidemiology (SRBE), Radiobiology and Radiopathology Research Laboratory (L3R), Fontenay-aux-Roses, France; 2 Department of Pathology, Rouen University Hospital, Rouen, France; 3 Department of Molecular, Cell, and Developmental Biology, University of California, California, USA

## Abstract

The current study evaluated the role of Hey2 transcription factor in radiation-induced endothelial-to-mesenchymal transition (EndoMT) and its impact on radiation-induced tissue damage in mice. Phenotypic modifications of irradiated, Hey2 siRNA- and Hey2 vector plasmid-transfected human umbilical vein endothelial cells (HUVECs) resembling EndoMT were monitored by qPCR, immunocytochemistry and western blots. Subsequently, in mice, a Cre-LoxP strategy for inactivation of Hey2 specifically in the endothelium was used to study the biological consequences. Total body irradiation and radiation proctitis were monitored to investigate the impact of conditional Hey2 deletion on intestinal stem cells and microvascular compartment radiosensitivity, EndoMT and rectal damage severity. We found that EndoMT occurs in irradiated HUVECs with concomitant Hey2 mRNA and protein increase. While Hey2 silencing has no effect on radiation-induced EndoMT *in vitro*, Hey2 overexpression is sufficient to induce phenotypic conversion of endothelial cells. In mice, the conditional deletion of Hey2 reduces EndoMT frequency and the severity of rectal tissue damage. Our data indicate that the reduction in mucosal damage occurs through decline in stem/clonogenic epithelial cell loss mediated by microvascular protection. EndoMT is involved in radiation proctitis and this study demonstrates that a strategy based on the reduction of EndoMT mitigates intestinal tissue damage.

## Introduction

About half of cancer patients undergo radiation therapy as part of their cancer treatment. Even though great advances have been made in treatment delivery techniques, allowing for better tumor targeting and sparing large volumes of healthy tissue, radiation exposure of significant volumes of normal intestine and rectum persists, limiting the chances of cancer cure and impacting on the patient’s quality of life post-treatment. Patients treated for abdominal-pelvic tumors may experience what is referred to as “pelvic radiation disease”^1^. Acute radiation damage is mainly due to the death of a significant proportion of epithelial stem cells and to compromised regeneration of differentiated epithelium, leading to mucosal ulceration and vascular compartment cell death and activation, which is responsible for a severe inflammatory reaction. The clinical consequences of acute damage concern 60 to 80% of patients and are expressed as abdominal pain and diarrhea. The acute phase can be followed by adverse tissue scarring resulting in gut fibrosis, with important associated morbidity such as dysmotility and malabsorption, but also fistulae and perforations of the most severe grades. Late adverse effects may occur from 3 months to several years following the end of radiation therapy and generally concern 5 to 10% of patients. Overall, external irradiation can be considered as a pro-inflammatory stimulus and the response of normal digestive tissue to ionizing radiation exposure is characterized by acute inflammation progressively evolving though chronic inflammation/excessive scarring^2^. Vascular abnormalities are consistently observed in injured healthy tissues of patients undergoing radiation therapy for cancer treatment, and endothelial injury has been described as a crucial event in the initiation and progression of radiation side effects in normal tissues^3^. Irradiated endothelial cells undergo apoptosis or may acquire a long-lasting procoagulant and antifibrinolytic phenotype with sustained immune cell recruitment, which participates in the development of tissue damage.

It has been known for several years now that adult cells retain a plastic capacity, as illustrated by the ability of epithelial and endothelial cells to undergo phenotypic conversion to mesenchymal-like cells via the processes of epithelial-to-mesenchymal transition (EMT) and endothelial-to-mesenchymal transition (EndoMT), respectively. During EndoMT, endothelial cells lose their endothelial markers (e.g. VE-cadherin, vWF, VCAM-1) and progressively gain expression of mesenchymal markers (e.g. α-SMA, SM22-α), while becoming motile. EndoMT was first discovered as a mechanism responsible for cardiac septation and formation of cardiac cushions and valves during embryogenesis^4^. EndoMT was also soon implicated in several systemic diseases, such as fibrodysplasia ossificans progressiva^5^ and systemic sclerosis^6^, and participates in tumor progression as an inducer of carcinoma-associated fibroblasts^7^. As for EMT, EndoMT can be subdivided into three main biological settings, namely organ development, tissue regeneration and scarring, and cancer progression and metastasis^8^. Data concerning EndoMT and tissue regeneration and scarring are relatively recent, and cells doubly positive for endothelial and mesenchymal markers, suggesting EndoMT, have been found in various contexts of tissue inflammation and fibrosis following different types of insult in the lung, heart and kidney^9–13^. Data concerning the putative role of EndoMT in gut damage are sparse, perhaps because it is complicated to obtain severe intestinal fibrosis in preclinical rodent models. Nevertheless, Rieder *et al*.^14^ identified EndoMT as a potential participant in human gut inflammation and in a preclinical model of colonic fibrosis in mice, demonstrating both *in vitro* and *in vivo* that inflammatory signals in the gut are able to trigger phenotypic conversion of endothelial cells to mesenchymal-like cells. We recently showed in a preclinical model of radiation proctitis in Tie2-GFP mice that radiation-induced tissue inflammation and scarring offer environmental conditions in favor of EndoMT, and that EndoMT is also present in human radiation proctitis^15^. Our next objective was to determine a putative pathway implicated in radiation-induced EndoMT and tissue damage, to offer new possibilities concerning the management of radiation injury to the gastrointestinal tract.

Previous mechanistic studies have highlighted the TGFβ and Notch signaling pathways as promoting EndoMT^16, 17^. The canonical Notch pathway is highly conserved in vertebrates and is essential in embryonic development, organogenesis and vascular remodeling in adults^18^. The role of Notch in EndoMT was first highlighted by Noseda *et al*., who demonstrated that overexpression of intracellular fragments Notch1IC and Notch4IC and of the ligand Jagged1 is sufficient to induce EndoMT in adult human macrovascular and microvascular cells^19^. Moreover, Notch overexpression in the endothelial compartment using a transgenic mouse model is associated with excessive endocardiac EndoMT, myocardial enlargement and the formation of hyperplastic valves, confirming the role of the Notch signaling pathway in EndoMT^17^. When activated, the Notch pathway carries out transactivation of target genes including transcriptional repressors of the Hes, Hers or Hey (hairy/enhancer of split-related with YRPW motif) genes^20^. Members of the Hers/Hey family are identified as the main effectors of the Notch pathway during development. Among Notch targets and together with Hey1, Hey2 has been implicated in embryogenesis, and Hey2 deficiency in mice displays defects in cardiovascular development^21^. Several studies have demonstrated the role of Hey2 in cardiac valve formation and especially in the EMT of endocardiac cells^22^. Whereas the role of the Notch target gene Hey2 in physiological EndoMT has been demonstrated, there are few studies on the putative role of Hey2 in stress-induced EndoMT^23, 24^.

Here we report that irradiation is a stimulus sufficient to induce EndoMT *in vitro* in human umbilical vein endothelial cells (HUVECs), associated with increased Hey2 mRNA and protein expression. Moreover, Hey2 overexpression is sufficient to induce phenotypic conversion of HUVECs to mesenchymal-like cells. Finally, conditional deletion of Hey2 in the endothelium in mice reduces EndoMT frequency and the severity of radiation-induced acute proctitis. Using a model of total body irradiation, we showed that Hey2 deletion in the endothelium reduced the number of apoptotic cells in the small intestinal stem cell compartment and increased surviving crypts. Immunostaining of plasmalemmal vesicle-associated protein suggests that Hey2 deletion may protect the endothelium, and consequently the epithelial stem cell compartment, from radiation damage. We thus propose reducing EndoMT as a possible strategy to mitigate radiation-induced damage to normal digestive tissue.

## Results

### Irradiation induces the endothelial-to-mesenchymal transition in HUVECs

**Figure 1:**
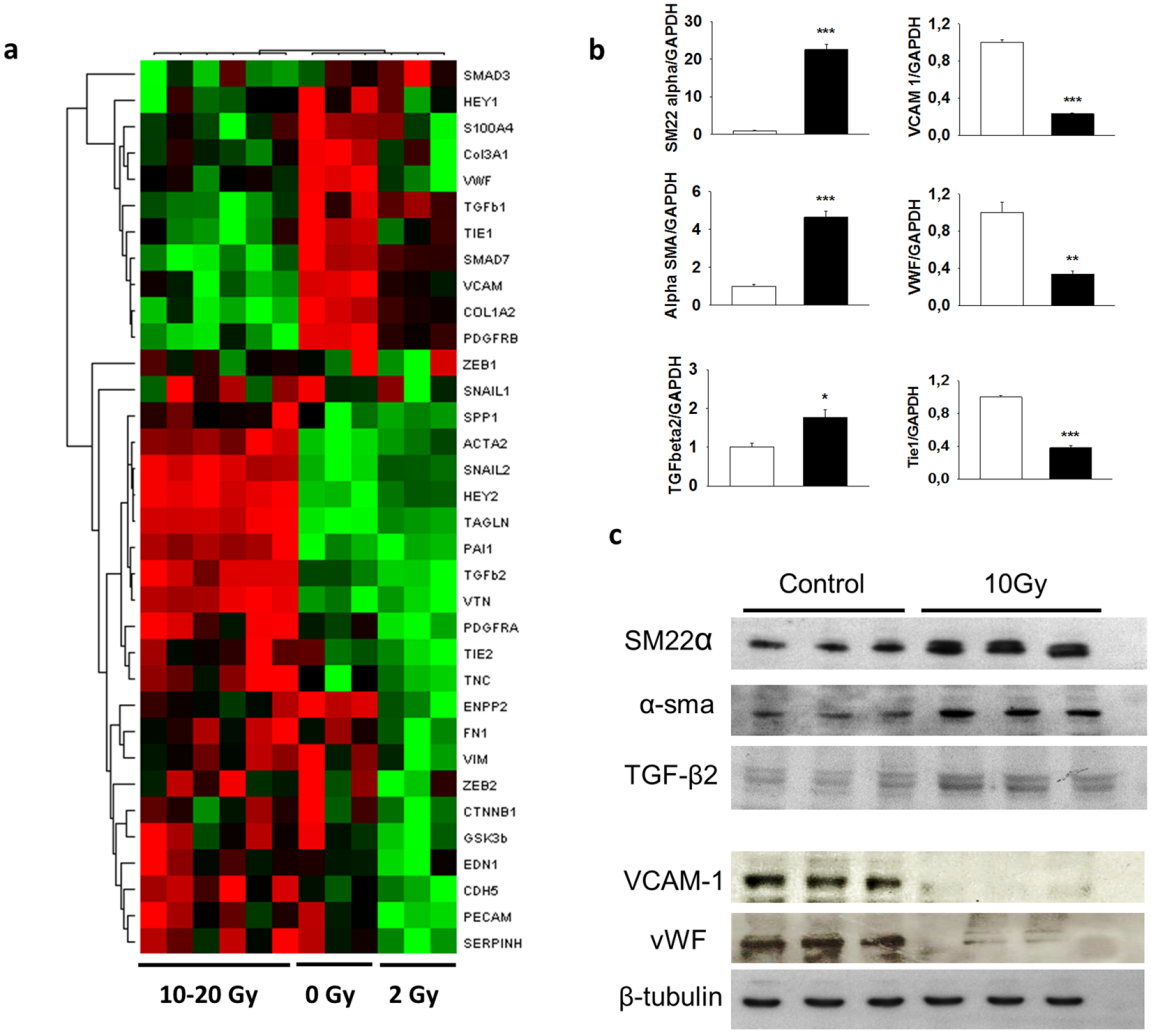
Irradiation induces phenotypic conversion of endothelial cells resembling EndoMT. (**a**) HUVECs were exposed to a single dose of 0, 2, 10 or 20 Gy and 34 genes related to the endothelial or mesenchymal phenotype and to the EndoMT process were measured by qPCR 7 days after radiation exposure. Hierarchical clustering shows different profiles of gene expression levels between control and irradiated cells. (**b**) Values of up- or down-regulation of expression of several endothelial and mesenchymal markers in irradiated HUVECs, 7 days after 10 Gy radiation exposure. (c) Confirmation of radiation-induced gene expression modifications at the protein level by western blot. Data are representative of three independent experiments performed in triplicate. *p < 0.05; **p < 0.01; ***p < 0.001.

**Figure 2:**
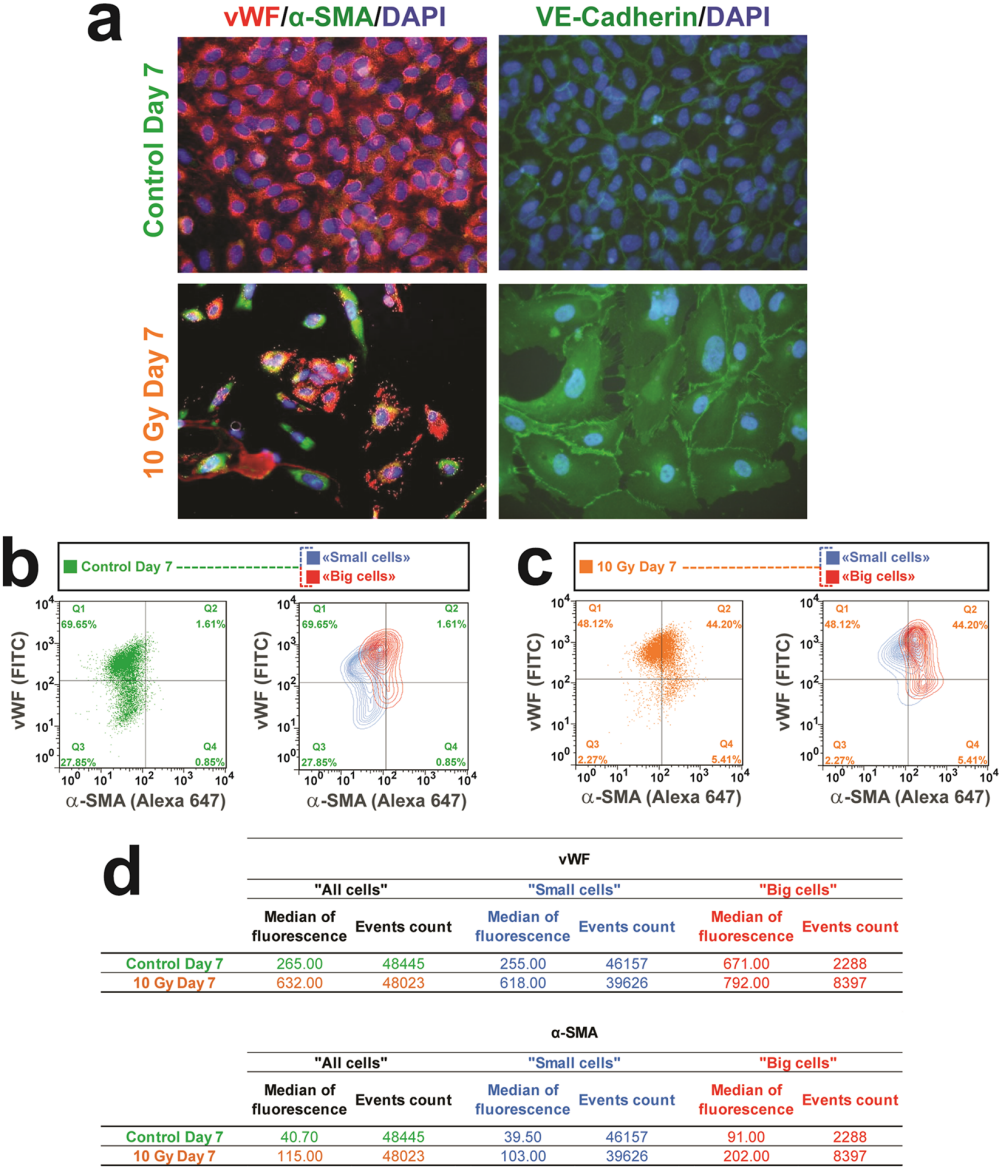
About 40% of HUVECs undergo phenotypic conversion. (**a**) Phenotypic characterization of 10 Gy-irradiated endothelial cells undergoing EndoMT by double immunostaining of vWF (red) and alpha-SMA (green) showing a yellow merging signal, which evidences co-expression of endothelial and mesenchymal markers of EndoMT. VE-Cadherin disorganization is also recognized as a feature of the EndoMT process. Representative images of 3 independent experiments. Original magnification x400. (**b** to **c**) Flow cytometry quantification of EndoMT by double immunostaining of vWF (FITC) and alpha-SMA (Alexa 647). Cut-offs for positive staining of both in control cells were defined at control day 7 (**b**) as below 2% (**b**, quarter Q2), showing co-expression marking EndoMT in around 44% of HUVECs on 10 Gy day 7 (**c**, quarter Q2). Median values of fluorescence peaks for each sample and both antibodies are given in the table (**d**). Data represent one representative experiment among three independent experiments.

The response of endothelial cells to radiation exposure is characterized by cell death and long-lasting phenotypic changes referred to as radiation-induced “activated” phenotype. To investigate whether these radiation-induced changes in the endothelial cell phenotype include EndoMT features, 90% confluent HUVECs were exposed to 0-, 2-, 10- or 20-Gy irradiation and monitored from day 4 to day 10 after exposure. We show that ionizing radiation induces a phenotypic conversion of HUVECs that resembles EndoMT. EndoMT is illustrated by heat map representation of expression levels of 34 different genes previously used to monitor EndoMT in human intestinal microvascular endothelial cells^15^ and related to endothelial or mesenchymal phenotypes and molecules involved in the EndoMT process 7 days after single doses of 2, 10 or 20 Gy exposure (Fig. 1a). Clustering occurs for 0, 2 and 10–20 Gy irradiated HUVECs. Given that 10 and 20 Gy irradiated cells clustered together, we decided to pursue the experiments on 10 Gy irradiated cells, to obtain satisfying phenotypic conversion without excessive cell death due to radiation exposure. The results show changes in expression levels of genes coding for proteins recognized as witnesses or inducers of the EndoMT process, such as increased α-SMA, SM-22α or TGF-β2, decreased vWF and VCAM1, or decreased Tie1 expression (Fig. 1b), whose deficiency has been shown to induce EndoMT^25^. Radiation-induced fold changes of several genes are dose-dependent (Supplementary Fig. 1). The global phenotypic switch at the mRNA level is conserved when cells are exposed to fractionated irradiation (20 Gy administered as 2 Gy daily x10, with a weekend break) as shown in Table 1. Radiation-induced changes in mRNA expression were confirmed at the protein level, with increased expression of mesenchymal markers SM-22α and α-SMA and reduced protein levels of the endothelial markers VCAM-1 and vWF, thus confirming EndoMT (Fig. 1c). To visualize phenotypic conversion of irradiated endothelial cells, we performed co-immunostaining of vWF and α-SMA (Fig. 2a). While control cells showed consistent vWF immunoreactivity (red), the irradiated cell population, 7 days after 10 Gy exposure, appeared heterogeneous, with sub-populations of vWF^+^ (red), α-SMA^+^ (green) and vWF^+^/α-SMA^+^ cells (yellow merging signal). Finally, VE-cadherin immunostaining revealed alterations in its distribution, with the appearance of cytoplasmic staining, a feature of EndoMT^26^.

**Table 1:** Gene expression profiles marking EndoMT in HUVECs after single dose or fractionated radiation exposure. HUVECs were exposed to 20 Gy administered in a single fractionated dose using 2 Gy per day/5 days a week with weekend break. Data are representative of 3 experiments for the single dose and one experiment for the fractionated dose, both done in triplicate and expressed as means ± sem. *p < 0.05; **p < 0.01; ***p < 0.001.

Gene (protein)	20 Gy single dose Day 7	20 Gy fractionated dose Day 7 post-last fraction
ACTA2 (alpha-SMA)	5.64 ± 0.85**	2.45 ± 0.22**
HEY2	9.57 ± 0.43***	2.64 ± 0.53*
SERPINE 1 (PAI-1)	1.77 ± 0.06***	1.98 ± 0.24*
TAGLN (SM22-alpha)	24.42 ± 2.36***	14.72 ± 1.65**
TGFbeta 2	1.77 ± 019*	2.39 ± 0.27*
TNC	4.32 ± 0.05	348.00 ± 17.79***
VTN	5.70 ± 0.9**	6.62 ± 0.35***
TIE1	0.42 ± 0.06***	0.50 ± 0.05**
TIE2	0.87 ± 0.04*	0.53 ± 0.05**
vWF	0.26 ± 0.03**	1.29 ± 0.10

### About 40% of HUVECs undergo phenotypic conversion to mesenchymal-like cells

Information on the proportion of cells undergoing EndoMT in the different published models is often missing, and may depend on the stimulus applied to induce phenotypic conversion. Given that not all cells exhibit phenotypic conversion, as illustrated by immunostaining experiments, flow cytometry was used to corroborate immunocytochemistry experiments, but also to estimate more precisely the percentage of α-SMA^+^ cells in the total HUVEC population. Prior to this, isotype controls were used to check the specificity of the staining, showing and testifying to the absence of interference or false-positive overlap signals (Supplementary Fig. 2). After exclusion of dead cells and debris from the analysis (Supplementary Fig. 3a and b), both FSC/SSC bi-parametric representation (Supplementary Fig. 3c–e) and FSC histogram representation (Supplementary Fig. 3f) clearly showed a shift of the size of the whole cell population in irradiated HUVECs compared with controls. A size cut-off of “small cells” versus “big cells” around 5% was determined for gated events in control cells in order to discriminate small cells from big cells. Seven days after 10 Gy irradiation, the proportion of big cells was 20%, 4 times more than the percentage observed in non-irradiated HUVECs.

Regarding α-SMA/vWF bi-parametric analyses, we used for the whole living cell population an α-SMA^+^/vWF^+^ cut-off below 2% for non-irradiated HUVECs (Fig. 2b, left panel, square Q2), this proportion of SMA^+^/vWF^+^ living cells representing around 44% for irradiated HUVECs (Fig. 2c, left panel, square Q2), showing that whereas α-SMA expression is virtually absent in control cells, around 44% of irradiated cells express α-SMA. Based on the cell size cut-off, α-SMA/vWF bi-parametric analysis was performed on small cells and big cells both for non-irradiated HUVECs (Fig. 2b, right panel) and for 10 Gy irradiated HUVECs (Fig. 2c, right panel). Globally, both small and big cells acquire α-SMA expression, big cells exhibiting, however, higher α-SMA staining than small ones, suggesting that EndoMT may be associated with an increase in cell size after radiation exposure. All these data confirm that radiation exposure of confluent HUVECs induces 7 days post-exposure changes in cell shape and mesenchymal marker expression, occurring in about 40% of the cell population at this time point.

### The endothelial-to-mesenchymal transition is associated with Hey2 overexpression in irradiated HUVECs

**Figure 3:**
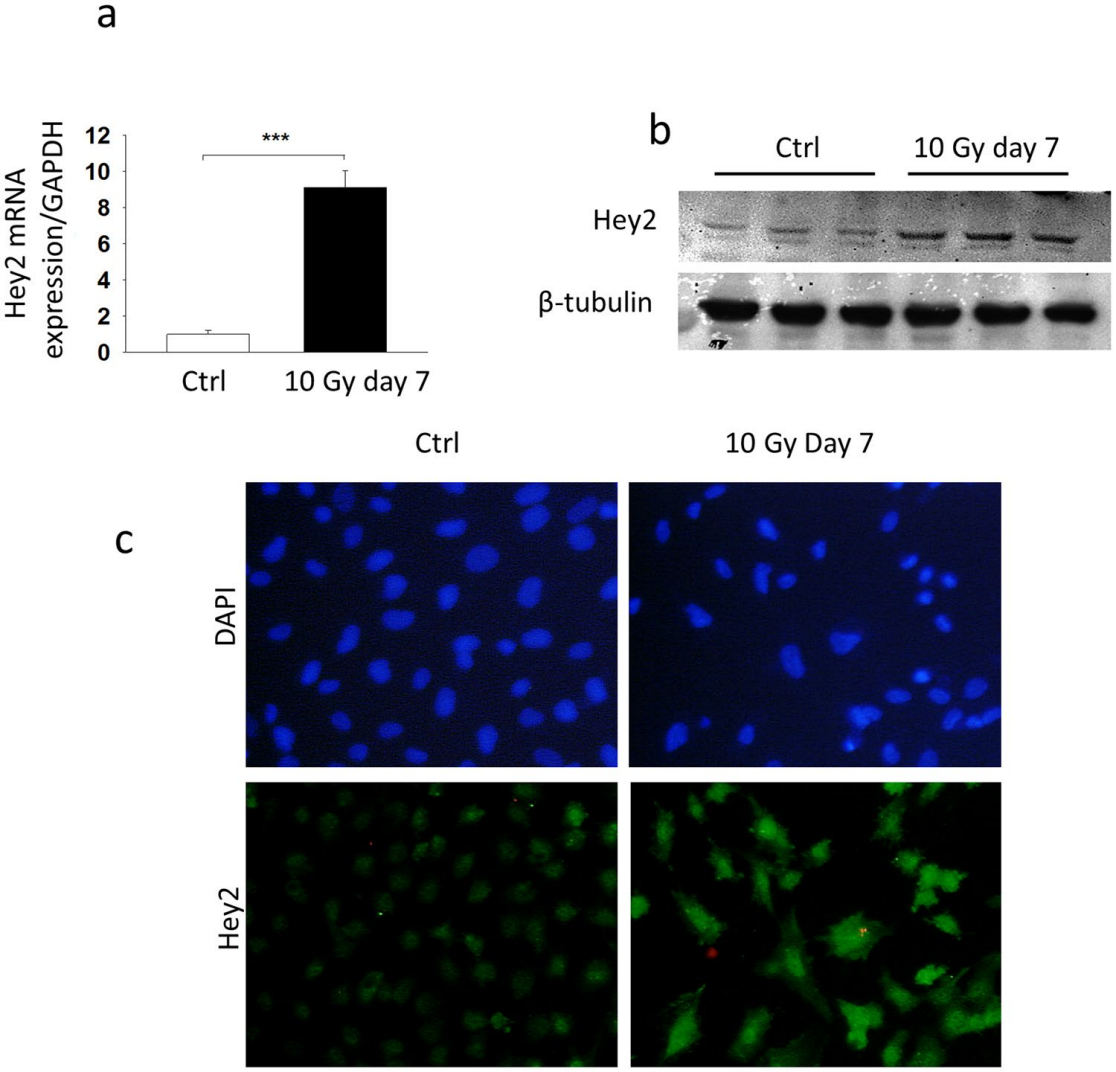
Radiation-induced EndoMT in HUVECs occurs together with Hey2 overexpression. (**a**) Hey2 mRNA levels in irradiated HUVECs were assessed by qPCR 7 days after 10 Gy exposure. (**b**) Western blotting for Hey2 protein expression in irradiated HUVECs, 7 days after radiation exposure to 10 Gy. (**c**) Hey2 immunostaining in HUVECs 7 days after 10 Gy exposure. Data are representative of three independent experiments performed in triplicate. ***p < 0.001.

EndoMT was initially discovered as an essential mechanism for heart development, with an important role played by the Notch pathway in primitive cardiac tube septation and the formation of cardiac valves^19^. Hairy and Enhancer of Split (HES)-related genes together with YRPW motif (Hey) genes (Hey1, Hey2 and HeyL) are the main target genes of the canonical Notch signaling pathway. Inactivation of *Hey2* results in severe embryonic defects in heart development, whereas deficiency in *Hey1* alone does not^27^. Based on bibliographic data, Hey2 was included in the set of genes related to EndoMT we chose for mRNA expression measurements and Hey2 appeared as an up-regulated gene in the hierarchical clustering (Fig. 1a). In accordance with previous studies relating to Hey2 and EndoMT in embryonic development, we demonstrated that EndoMT induced by exogenous stress is also associated with Hey2 mRNA and protein overexpression, as confirmed by western blot and immunostaining (Fig. 3). Radiation-induced Hey2 mRNA overexpression is dose-dependent (Supplementary Fig. 4a), maintained over time until at least 10 days post-exposure and independent of culture plastic coating. Conversely, Hey1 expression level is decreased by radiation exposure (Supplementary Fig. 4b).

### Hey2 overexpression using expression vector transfection in HUVECs is sufficient to induce EndoMT

When we observed that radiation-induced phenotypic conversion of HUVECs was associated with Hey2 overexpression, we decided to test the ability of Hey2 siRNA to influence radiation-induced EndoMT *in vitro*. HUVECs were transfected with Hey2 siRNA to inhibit Hey2 mRNA expression and exposed to 10 Gy irradiation. Despite marked reduction in mRNA expression levels in transfected cells even after irradiation (>90%), Hey2 inhibition had no effect on radiation-induced α-SMA and TGFβ2 overexpression or on Tie1 down-regulation (Supplementary Fig. 5). SM22-α expression was significantly reduced and expression of VCAM-1 and of vWF returned to control values in the presence of Hey2 siRNA, but also in the presence of non-targeting siRNA, probably reflecting a transfection-related effect. Overall, these results show that increased Hey2 expression levels *in vitro* are dispensable for radiation-induced EndoMT.

**Figure 4:**
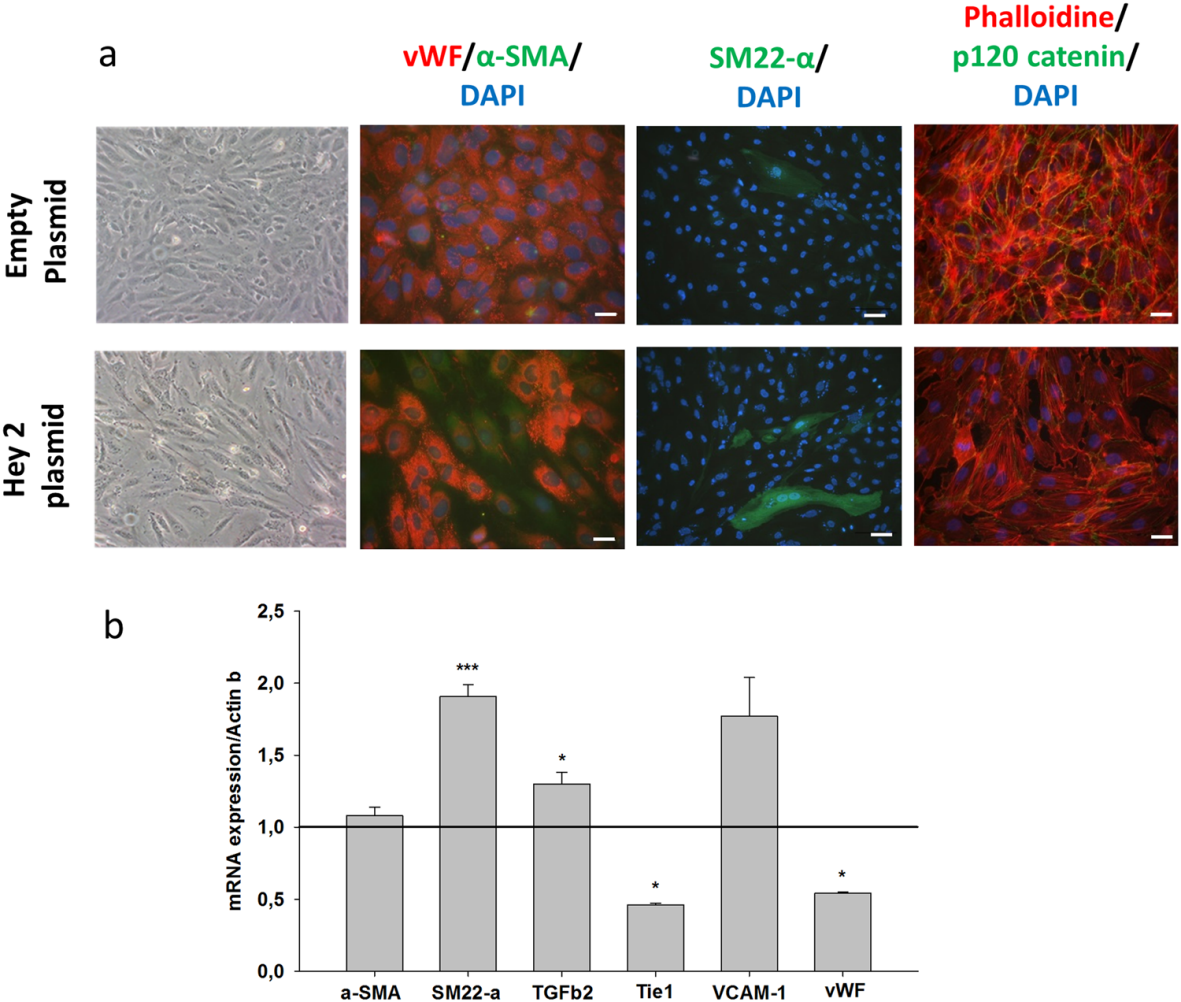
Hey 2 overexpression is sufficient to induce phenotypic conversion of endothelial cells to a mesenchymal phenotype. HUVECs were transfected with pCMV6-HEY2-Myc-DDK plasmid expression vector or with pCMV6-AC-HA-His as a control. Three days after transfection, some cells were spindle-shaped, lost their vWF expression while acquiring alpha-sma and SM22-alpha expression, and lost some intercellular connections via p120 catenin. Optical observation x400; immunostaining: bar = 50 µm, except for SM22-α bar = 100 µm. (**b**) mRNA expression levels measured by qPCR in Hey2 expression plasmid-transfected HUVECs compared with HUVECs transfected with empty plasmid (retrieved to 1, bar), 72 h after cell transfection. *p < 0.05; ***p < 0.001. Data are representative of two independent experiments performed in triplicate, and values are the mean ± sem.

We next decided to investigate if Hey2 by itself was able to induce EndoMT *in vitro*. Cell transfection with Hey2 plasmid expression vector induced EndoMT (Fig. 4a). Transfected cells acquired a typical spindle-shaped morphology, and showed decreased vWF and increased SM22-α immunoreactivity. Finally, irradiated cells exhibited partial loss of intercellular junctions, as illustrated by p120-catenin immunostaining, a feature of phenotypic conversion of endothelial cells to mesenchymal-like cells. Figure 4b shows the mRNA expression levels of several genes measured in Hey2 plasmid-transfected cells compared with empty plasmid-transfected cells. Hey2 overexpression in HUVECs was associated with increase in SM22-α and TGFβ2 expression and reduced expression of Tie1 and vWF, as observed after radiation exposure. Conversely, and as opposed to radiation-induced EndoMT, expression of VCAM-1 and α-SMA was unchanged. These data show that overexpression of Hey2 is sufficient to induce phenotypic conversion of HUVECs to a mesenchymal-like phenotype, albeit with some differences compared with radiation-induced EndoMT.

### Human radiation proctitis is associated with increased Hey2 immunoreactivity in injured tissues

**Figure 5:**
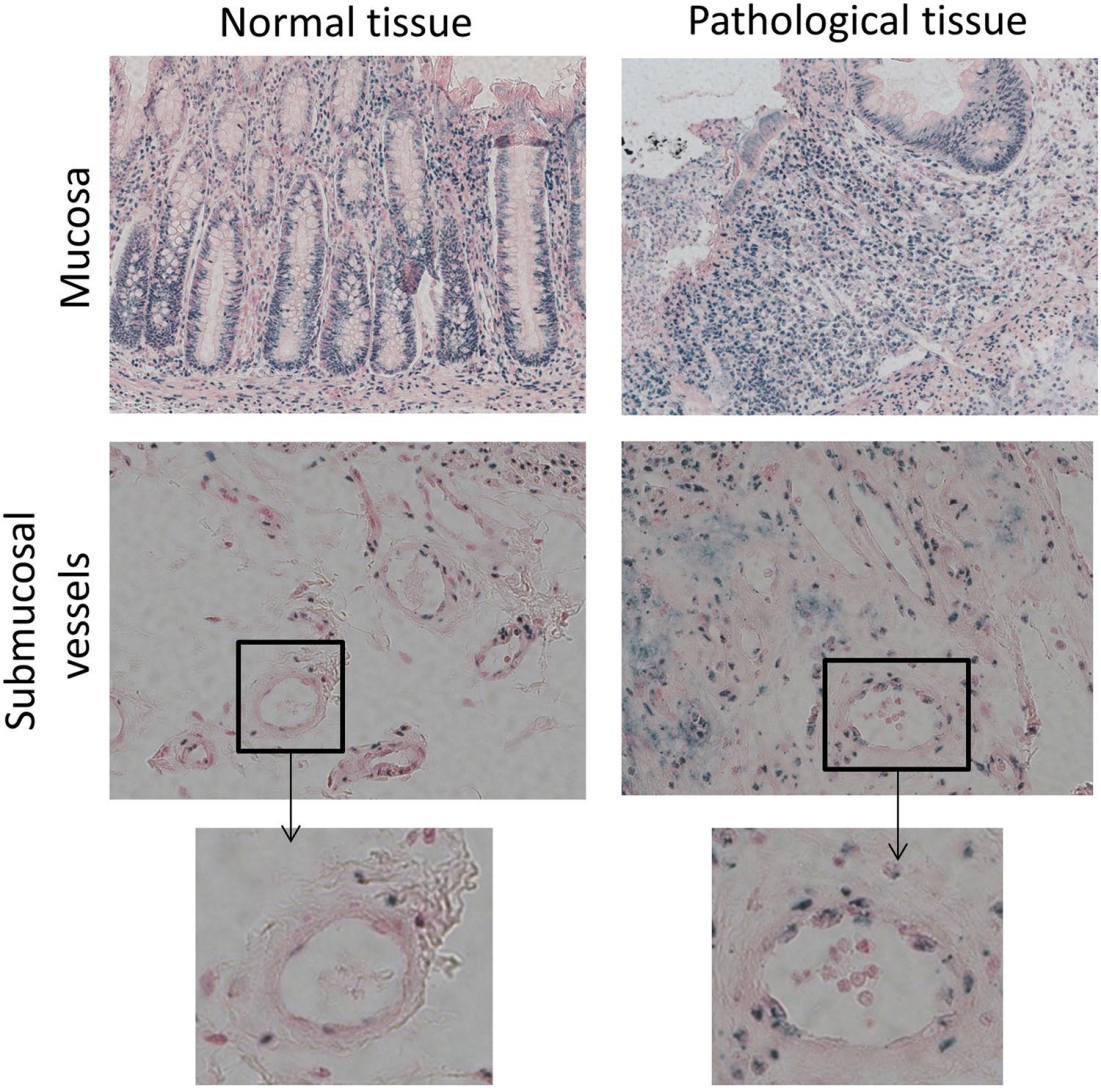
Hey2 immunoreactivity in the endothelial compartment is increased in human radiation proctitis. Hey2 immunostaining was performed in human rectal tissues (pictures of mucosa and submucosa are presented) following pre-operative radiation therapy for rectal adenocarcinoma at a total dose of 45 Gy. Surgical resection occurred 5–7 weeks after radiation therapy taking rectal tissue within the irradiation field near the tumor bed (pathological tissue) and in margins (normal tissue) outside the irradiation field. Insert: submucosal vessel magnification detailing Hey2 immunoreactivity in endothelial cells. Original magnification x200.

Radiation exposure offers microenvironment conditions favoring EndoMT, such as cell apoptosis, oxidative stress, barrier rupture, tissue inflammation, fibrosis and hypoxia, as demonstrated *in vitro* or in different models of trauma^14, 28–30^. We previously showed that EndoMT was present in human radiation proctitis, especially in severely injured/inflamed tissues^15^. Normal rectal tissue immunostaining revealed a general nuclear expression of Hey2 in all cell types including epithelial and endothelial cells. Pathological tissue showed invasion of Hey2-positive immune cells and a possible increase in endothelial cells of nuclear immunoreactivity for Hey2 in several submucosal vessels (Fig. 5). Nevertheless, these results should be interpreted cautiously, given that a lot of submucosal vessels in damaged tissues showed only slight Hey2 staining.

### Conditional deletion of Hey2 in the endothelium reduces endothelial-to-mesenchymal transition frequency associated with preclinical acute radiation proctitis

**Figure 6:**
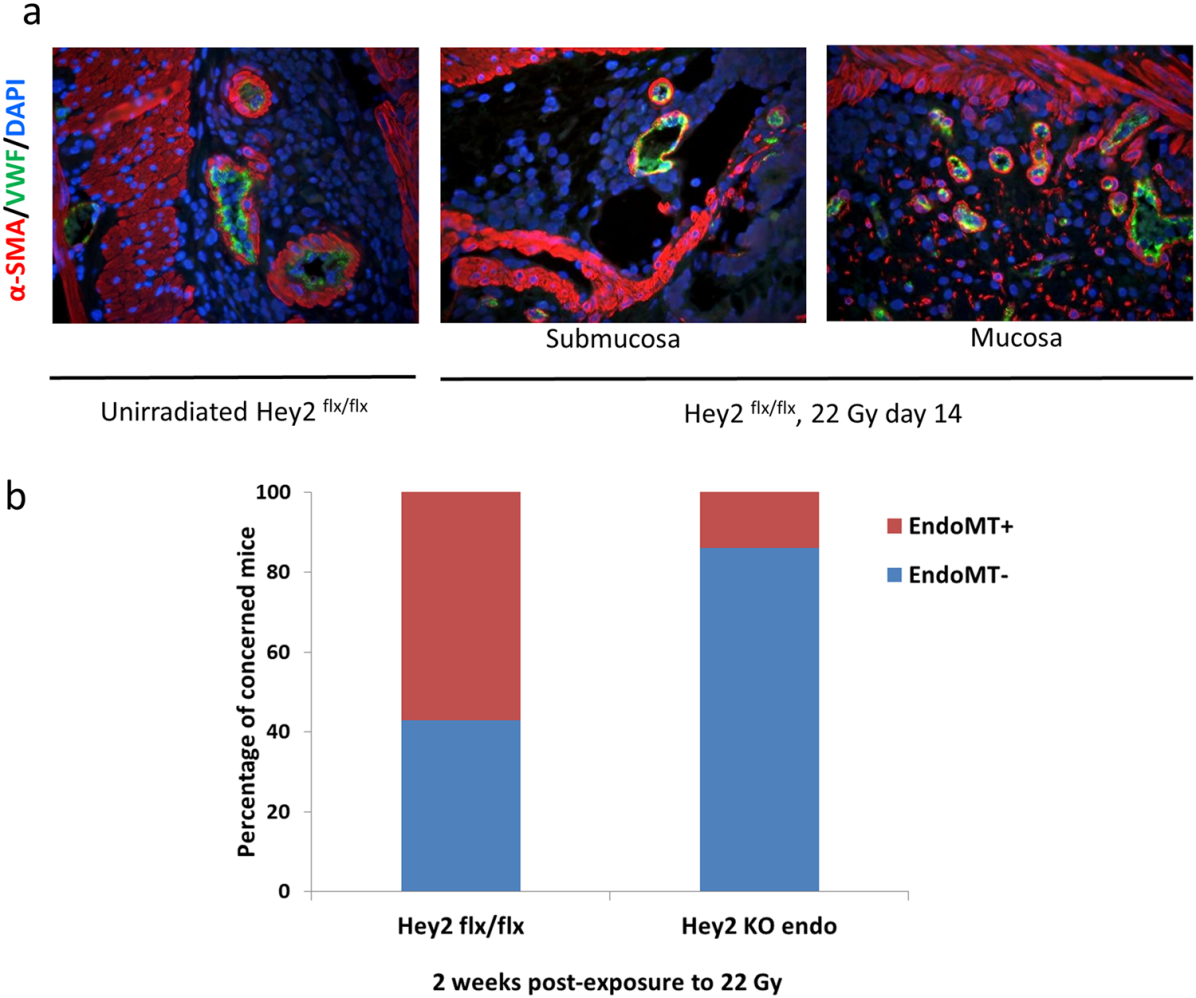
Conditional Hey2 knock-out in the endothelium reduces EndoMT frequency in injured colorectum. Hey2^flx/flx^ and Hey2KO^endo^ mice were exposed to 22 Gy in the colorectal area. (**a**) Representative pictures of vWF/α-sma co-immunostaining showing no merging signal in unirradiated tissues, and EndoMT figures (yellow merging signal marking co-expression) observed by confocal microscopy in the mucosa as well as in the submucosa of 22 Gy irradiated tissues. Co-expression was observed in both mouse strains. Original magnification x400. (**b**) Hey2 conditional deletion in the endothelium reduced the percentage of mice concerned with EndoMT 14 days after radiation injury. Hey2^flx/flx^ n = 6; Hey2KO^endo^ n = 7.

The Notch signaling pathway and its main downstream target gene Hey2 have been implicated in the EndoMT process during cardiac development and in endothelial cells *in vitro* ^8^, but to our knowledge there are currently few findings regarding the role of Hey2 in stress-induced EndoMT. Following *in vitro* observations of overexpression of Hey2 mRNA and protein in irradiated endothelial cells, we decided to inactivate Hey2 specifically in the endothelial compartment using the Cre-LoxP strategy, to avoid defects in cardiac development associated with Hey2 knock-out^21^. To do this, we crossbred Hey2^flx/flx^ mice with VE-CadCre^+/+^ mice to obtain Hey2^flx/flx^/Ve-CadCre^−/−^ mice as controls (Hey2^flx/flx^) and Hey2^flx/flx^/Ve-CadCre^+/−^ mice (Hey2KO^endo^) in which Hey2 expression is inactivated specifically in the endothelial compartment (Supplementary Fig. 6). Both were submitted to 22 Gy colorectal irradiation. The first step was to investigate whether Hey2 deletion in endothelial cells lessened radiation-induced EndoMT in our preclinical model of radiation proctitis. Co-immunostaining of vWF and α-SMA evidenced transitioning endothelial cells in irradiated tissues by the co-expression of both markers (Fig. 6a). EndoMT occurred in the submucosal as well as in the mucosal vessels, as already observed in this model^15^. Both Hey2^flx/flx^ and Hey2KO^endo^ mice exhibited EndoMT, albeit with a reduced percentage of mice exhibiting EndoMT in the Hey2KO^endo^ group (Fig. 6b), demonstrating that Hey2 deletion in endothelial cells is an effective strategy to reduce the frequency of radiation-induced EndoMT in digestive tissues.

### Reducing EndoMT frequency impacts the severity of acute and late preclinical radiation proctitis

**Figure 7:**
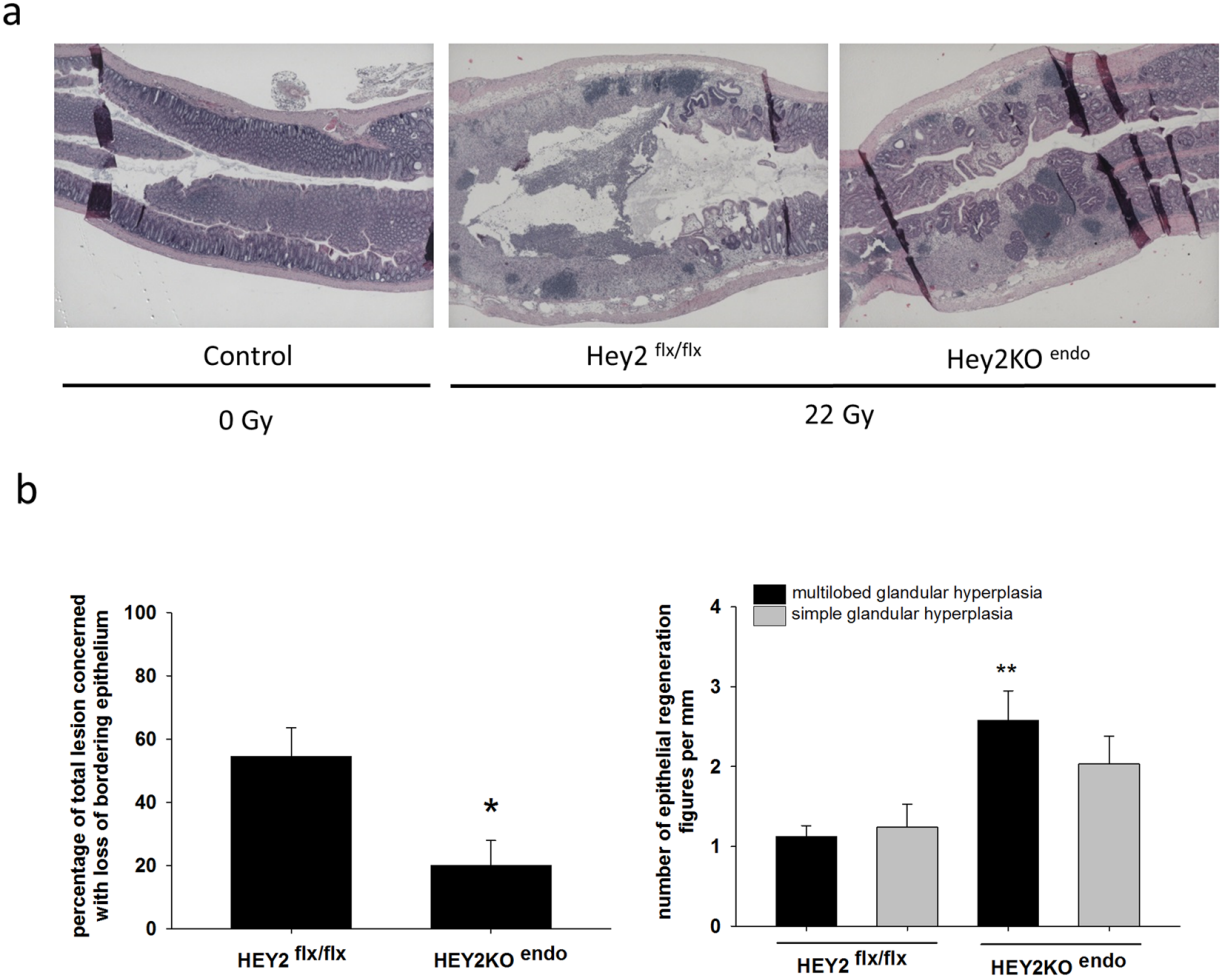
Conditional Hey2 deletion in the endothelium mitigates radiation-induced acute tissue damage. (**a**) HES staining of control colorectum and irradiated colorectum of Hey2^flx/flx^ and Hey2KO^endo^ mice 14 days post-exposure. Original magnification x40. (**b**) The percentage of total lesions demonstrating loss of bordering epithelium (ulceration) and multilobed and simple glandular reactive epithelial hyperplasias were counted for each mouse over a total injured area length between 4 and 9 mm 14 days post-injury. Hey2^flx/flx^ n = 6; Hey2KO^endo^ n = 7.

Exposure of mouse rectum to a single 22 Gy dose of ionizing radiation resulted in severe acute (14 days) mucosal damage, with crypt rarefaction, severe mucosal inflammation and ulceration. Ulceration is defined as the total loss of bordering epithelium. Ulceration is a sign of severity because the absence of bordering epithelium exposes underlying tissues to luminal contents and favors severe intestinal transmural inflammation. The 14-day time point in this model is also characterized by reactive hyperproliferation of surviving crypts, demonstrating high cell density and often multilobed glands that sometimes resemble adenocarcinoma (Fig. 7a). Glandular hyperplasia, as opposed to epithelial ulceration, is a sign of tissue scarring. Epithelial cell production by hyperplastic crypts to recover the bordering epithelium is a crucial step in repair of injured intestinal tissue. To evaluate the severity of radiation damage in both Hey2^flx/flx^ and Hey2KO^endo^ mice, we measured the percentage of total lesions concerned with complete loss of bordering epithelium and the number of simple and multilobed glandular hyperplasias. Acute tissue protection in Hey2KO^endo^ mice was characterized by a lower percentage of mucosa concerned with bordering epithelial loss and more multilobed glandular hyperplasias (Fig. 7b), suggesting reduced acute tissue damage.

**Figure 8:**
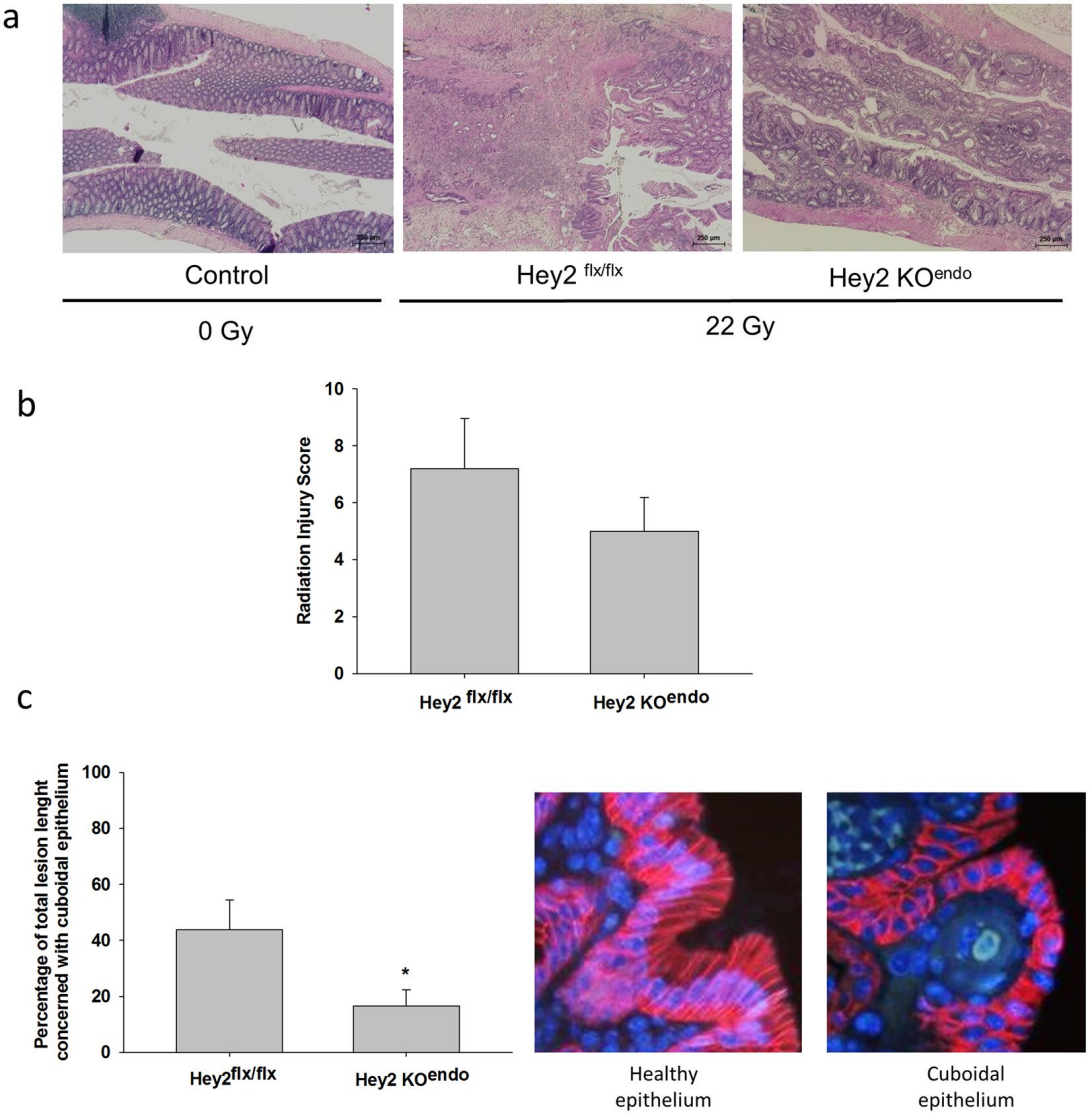
Acute injury mitigation by conditional deletion of Hey2 reduces the severity of late epithelial damage. (**a**) HES staining of control and irradiated rectal tissues 7–8 weeks following localized radiation exposure to 22 Gy showing radiation damage observed in Hey2^flx/flx^ and Hey2KO^endo^ mice. (**b**) A semi-quantitative radiation injury score was assigned to irradiated tissues from both strains. Original magnification x40. (**c**) the percentage of bordering epithelium with cuboidal epithelial cells marking epithelial damage was measured on each HES- and p120 catenin-stained (pictures) tissue section. Original magnification x400. Hey2^flx/flx^ mice n = 9; Hey2KO^endo^ mice n = 12. *p < 0.05.

Exposure of the rectum to high single doses of ionizing radiation is known to generate late effects with a strong consequential component, i.e. the severity of late damage is highly influenced by the severity of acute injury. Given that Hey2 deletion in the endothelial compartment lessened acute radiation proctitis, we aimed to determine whether this has an impact on late rectal injury. Late damage was characterized by mucosal ulcer healing, but also by persistent severe epithelial atypia, crypt hyperplasia, mucosal collagen deposition and moderate vascular dystrophy. HES staining in Fig. 8a shows visibly reduced severity 7–8 weeks post-irradiation in Hey2KO^endo^ compared with Hey2^flx/flx^ mice, but the Radiation Injury Score (RIS, Fig. 8b) failed to reach statistical significance. The RIS consists of several parameters related to all intestinal wall compartments, thus minimizing the impact of mucosal damage score on the total score applied. We investigated more precisely late mucosal damage by measuring the percentage of total lesions affecting healthy bordering epithelium (well-oriented/elongated columnar cells with basal nucleus) and those indicating atypical epithelium (cuboidal epithelial cells with centered nucleus, pictures in Fig. 8c). These measurements were done using HES staining and p120-catenin immunofluorescent staining. Hey2KO^endo^ mice showed a lower percentage of total lesions affecting cuboidal/atypical epithelium than Hey2^flx/flx^ mice (Fig. 8c). These data may indicate lessened severity of late radiation proctitis in Hey2KO^endo^ mice.

### Conditional deletion of Hey2 in the endothelium protects the intestinal stem cell compartment from radiation damage

**Figure 9:**
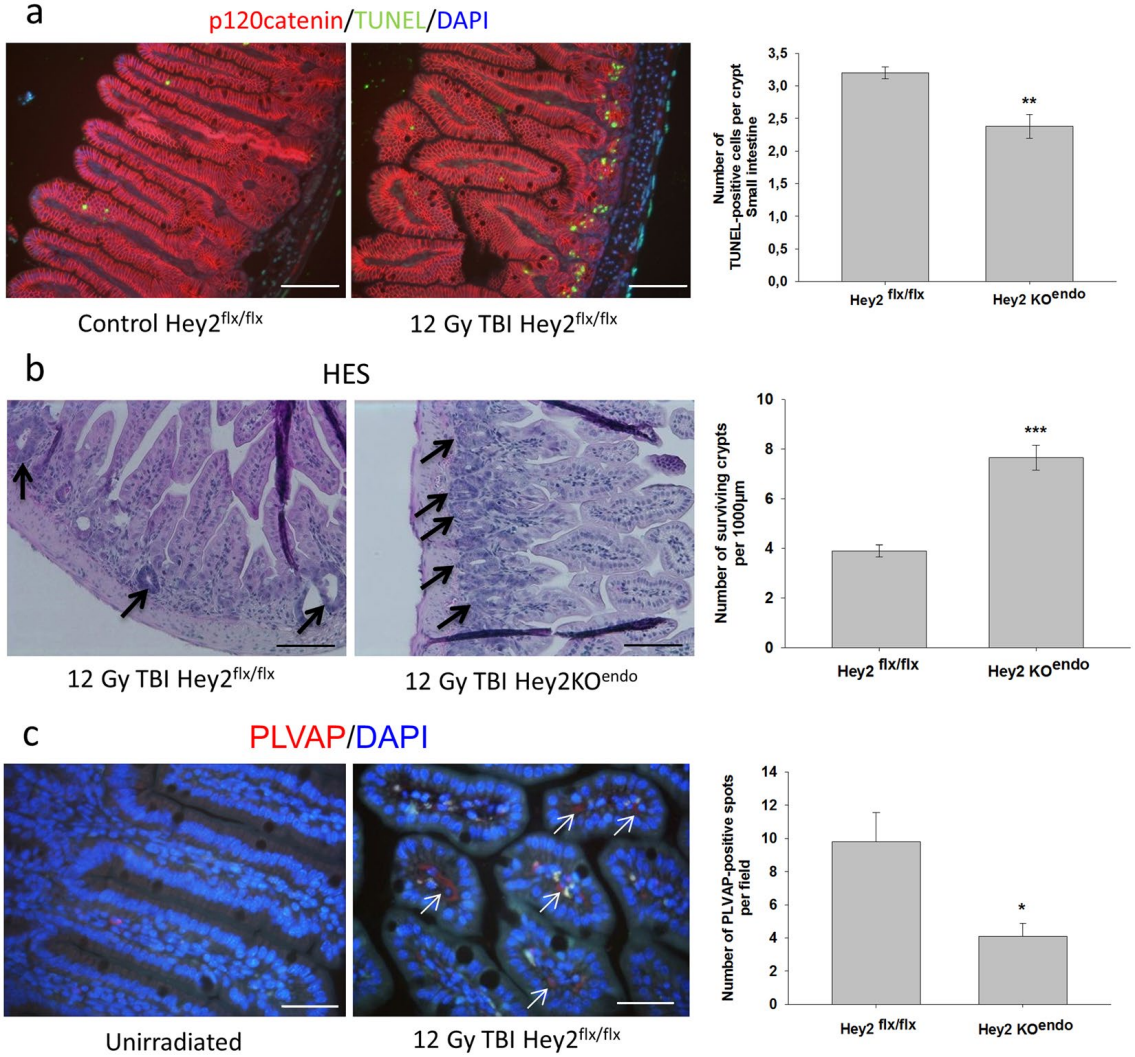
Conditional Hey2 deletion in the endothelium protects epithelial stem cells and endothelial compartments from radiation damage. (**a**) Hey2^flx/flx^ and Hey2KO^endo^ mice were exposed to 12 Gy total body irradiation (TBI) and TUNEL/p120-catenin co-immunostaining was performed 24 h post-irradiation to monitor apoptosis of crypt epithelial cells. The number of apoptotic crypt cells was determined in the small intestine in a total of 50 crypts per slide. (**b**) HES staining of small intestine tissue was performed 3 days after total body irradiation. Surviving crypts were identified as surviving irradiation based on their histological appearance, i.e. undilated and with at least 10 well-stained epithelial cells and 1 Paneth cell. Surviving crypts were counted over a total tissue length between 1900 and 2600 µm and related to 1000 µm in both Hey2^flx/flx^ (n = 6) and Hey2KO^endo^ mice (n = 3). **p < 0.01; ***p < 0.001. Bar = 100 µm. (**c**) Plasmalemmal Vesicle-Associated Protein-1 (PLVAP, red) immunostaining of small intestine tissue three days post-12 Gy TBI. Bar = 50 µm. PLVAP immunoreactivity was rare in control tissues, but increased dramatically after irradiation. The number of PLVAP-positive spots was thus quantified in 5 different fields per slide in irradiated small intestine in both strains. Hey2^flx/flx^ n = 4; Hey2KO^endo^ n = 3. *p < 0.05.

Total body irradiation is a model frequently used to investigate the role of several agents in the regenerative capacity of the small intestinal stem cell compartment^31^. We saw that Hey2 endothelial deletion reduced acute mucosal ulceration. Ulceration is defined as the loss of bordering epithelium, due to radiation-induced insult to/sterilization of the stem/progenitor crypt compartment and compromised epithelial regeneration. To assess intestinal crypt response to ionizing radiation, we exposed Hey2^flx/flx^ and Hey2KO^endo^ mice to 12 Gy total body irradiation and monitored small intestinal stem cell apoptosis (24 h post-exposure) and the number of surviving crypts at 3 days, indirectly reflecting the number of surviving stem cells. TUNEL/p120 catenin double immunostaining revealed radiation-induced cell apoptosis in the stem and transit/amplifying compartments as exemplified by pictures obtained in control and 12 Gy total body-irradiated Hey2^flx/flx^ mice (Fig. 9a). The number of TUNEL-positive cells per crypt was significantly reduced in Hey2KO^endo^ mice compared with Hey2^flx/flx^ mice (Fig. 9a), suggesting intestinal crypt compartment protection by conditional Hey2 deletion in the endothelium. The severity of intestinal radiation damage may be further evaluated by counting the surviving crypts. Three to four days post-irradiation, the number of crypt-like foci of surviving epithelial cells can be counted on histological/HES-stained tissue sections based on their morphological appearance, i.e. no base dilatation and presenting at least 10 well-stained epithelial cells and one Paneth cell. The number of surviving crypts at 3 days post-exposure was significantly higher in Hey2KO^endo^ mice than in Hey2^flx/flx^ mice (Fig. 9b), confirming the epithelial protection afforded by the conditional deletion of Hey2 in endothelial cells.

The next question was how an endothelial-specific Hey2 deletion influences intestinal stem compartment response to radiation exposure. Our hypothesis was that protection of crypt cells in Hey2KO^endo^ mice occurred via protection of endothelial cells from radiation damage. Endothelial protection, and especially reduced endothelial apoptosis, has been shown to protect small intestinal crypts from radiation damage. Radiation-induced apoptosis of endothelial cells has been observed in the chorion of small intestinal villi. Probably because the mucosal microvasculature is more difficult to observe, we were unable to see any radiation-induced endothelial apoptosis in irradiated rectum 5 h post-exposure. We then decided to use Plasmalemmal Vesicle-Associated Protein-1 (PLVAP/PV-1) immunostaining. PLVAP is a transmembrane protein associated with the caveolae of fenestrated microvascular endothelia. PLVAP up-regulation is a marker of endothelial barrier disruption and endothelial activation, and is often used to monitor the state of the blood-brain barrier^32^. PLVAP immunostaining was performed in 22 Gy irradiated rectal tissues 2 and 7/8 weeks post-exposure, showing no difference from unirradiated tissues in which PLVAP immunostaining was virtually absent. We hypothesized that earlier time points following radiation exposure may be more informative and performed PLVAP immunostaining 24 h and 3 days after 12 Gy total body irradiation in rectal and small intestinal tissues. Whereas there was again no PLVAP positive signal at 24 h, the 3-day time point was characterized by a marked increase in PLVAP immunoreactivity (Fig. 9c). The number of PLVAP-positive spots was thus quantified in both mouse strains on small intestinal tissue sections to allow easier observation of microvascularization than in rectal sections. Conditional Hey2 deletion lessened the number of PLVAP-positive spots in small intestinal tissue sections (Fig. 9c), suggesting reduced endothelial barrier disruption in Hey2KO^endo^ mice.

## Discussion

The response of normal digestive tissue to radiation exposure is characterized by inflammation and fibrosis, a context known favor EndoMT. We recently demonstrated that EndoMT participates in tissue injury in radiation-induced proctitis in Tie2-GFP mice and is present in human radiation proctitis following rectal cancer treatment by radiation therapy^15^. This earlier report, however, did not provide information about possible mechanisms and molecular targets implicated in radiation-induced EndoMT. The present study demonstrates that Hey2, a transcriptional repressor and Notch pathway target, is implicated in radiation-induced EndoMT, and that its conditional deletion in the endothelial compartment efficiently reduces EndoMT and consequently the severity of tissue damage in our preclinical model of radiation-induced proctitis. This is the first report demonstrating that EndoMT may represent an interesting strategy to mitigate radiation-induced damage to healthy digestive tissues.

Adult endothelial cells possess plastic properties allowing phenotypic conversion to mesenchymal cells via different induction methods. The response of endothelial cells to radiation exposure is characterized by cell death, but also by the acquisition of a long-lasting activated phenotype responsible for now well-described radiation-induced vascular dysfunction. The endothelial compartment is present in normal tissues as well as in tumors, and advances in the radiobiology of endothelial cells may be determinant in understanding how tumors and healthy tissues respond to radiation therapy^3, 33^. EndoMT has been shown to belong to the various phenotypic changes observed after radiation exposure of endothelial cells, and was previously demonstrated in irradiated human aortic endothelial cells^30^, human pulmonary endothelial cells^29^ and human intestinal microvascular endothelial cells (HIMECs)^15^. Here we made the choice to use HUVECs as an *in vitro* model to explore possible candidate genes involved in radiation-induced EndoMT that may represent interesting molecular targets. The use of HUVECs is, of course, questionable, given that we had the opportunity to use HIMECs. Our objective in the present study was to obtain the more representative gene expression profile in primary cells obtained from a pool of patients rather that extracted from a particular one, and to work with less precious and easily transfectable cells. The ability of HUVECs to undergo phenotypic conversion to mesenchymal cells has been demonstrated using various stimuli^34, 35^ and here we show that ionizing radiation is another stimulus able to induce EndoMT in these human macrovascular cells, with a gene expression profile resembling the one obtained in irradiated HIMECs^15^. *In vivo* as well as *in vitro*, it is known that not all endothelial cells undergo EndoMT. *In vivo*, there are percentage estimates concerning the number of mesenchymal cells of endothelial origin in several models^10, 12, 13^, but the number of endothelial cells undergoing EndoMT within the vasculature *in vivo* remains unknown, even *in vitro* in the different situations published hitherto. In the present study we demonstrate that transitioning endothelial cells 7 days after radiation exposure to 10 Gy represent around 40% of total confluent HUVECs. However, we do not know if this percentage represents what happens *in vivo*. Finally, we observed that EndoMT especially concerned “big cells”, i.e. probably irradiated cells becoming senescent, with a characteristic increase in cell size^36^. Further work is needed to establish whether radiation-induced EndoMT shares common features with cellular senescence.

Study of gene expression revealed a marked and very reproducible increase in Hey2 expression levels, and we made the choice to investigate the role of Hey2 in radiation-induced EndoMT. Hey2 is a Notch pathway target acting directly as a transcriptional repressor and may activate some target genes by indirect mechanisms. The link between Notch and Hey2 is not exclusive, since Notch1^−/−^ mice still express Hey2 and transcription of Hey2 can be stimulated via the BMP9/Alk1 pathway independently of Notch activation in HUVECs^21, 37^. Data demonstrating the role of Hey2 in stress-induced EndoMT or even EMT are sparse and the present study is the first to show that Hey2 overexpression is sufficient to induce EndoMT in HUVECs, decreasing expression of vWF and Tie1 and increasing expression of SM22-α and TGFβ2. The absence of increase in α-SMA expression may be due to study timing. Nevertheless, this is not troublesome considering that the precise phenotype obtained in endothelial cells undergoing EndoMT may vary depending on the stimulus applied. This is well demonstrated by the literature, in which transitioning cells often have very different expression profiles. Unlike the majority of stimuli applied to endothelial cells to induce EndoMT in published studies, such as cytokines^14^, glucose^34^ or oxidative stress^38^, radiation exposure generates a highly complex cell response with cytokine secretion, oxidative stress or again cell death and senescence. This complexity may explain why transfection of HUVECs with Hey2 siRNA before irradiation failed to rescue EndoMT progression, suggesting the involvement of multiple cellular pathways.

Because transfection by itself may have deleterious effects and does not represent what happens *in vivo*, we decided to investigate the involvement of Hey2 in radiation-induced EndoMT in our preclinical model of radiation proctitis in mice. Given that Hey2 knock-out is associated with severe cardiac defects, we used the Cre-LoxP strategy to inactivate Hey2 specifically in the endothelial compartment. The specificity of endothelium recombination events in intestinal tissue was checked using ROSA26 reporter mice crossed with Ve-Cad-Cre mice in previous studies^15^. We used a preclinical model of high-dose localized exposure to the rectum to mimic the severe mucosal ulceration that may be encountered in rectal tissues of patients treated with radiation therapy. This model does not fit with the majority of radiation fractionation schedules used in humans until now, but the evolution of radiation therapy techniques, such as Stereotactic Body Radiation Therapy (SBRT) using ablative doses per fraction in the treatment of prostate tumors, makes this preclinical approach pertinent to the investigation of the possible mechanisms of radiation side effects in such a context.

In this study we show that conditional deletion of Hey2 in the endothelial compartment reduces EndoMT frequency and the severity of radiation proctitis. This enabled us to overcome a barrier to our understanding of the role of EndoMT in radiation proctitis compared with our previous work^15^, demonstrating here that reducing EndoMT has a direct impact on tissue damage severity, as demonstrated in mouse models of cardiac fibrosis or obstructive nephropathy^10, 39^. Reduced tissue damage in Hey2KO^endo^ mice in the present study was expressed as lessened mucosal ulceration. Ulceration results from compromised epithelial regeneration and damage to the gastrointestinal tract after radiation exposure and has been shown to result from the destruction of radiosensitive compartments such as crypt/stem and clonogenic epithelial cells and microvascular endothelium^31, 40^. We thus first explored radiation effects on the epithelial stem compartment and observed that Hey2 deletion in the endothelium preserved crypt function, which may explain reduced ulceration by more efficient epithelial cell production.

We currently do not know how conditional deletion of Hey2 in the endothelium protects the epithelial compartment. One hypothesis is preservation of microvascular endothelial integrity. To investigate the condition of the microvascular endothelium, we used PLVAP or PV-1 immunostaining. PLVAP belongs to the caveolae and diaphragmed fenestrae structures of fenestrae endothelia, which finely tune endothelial permeability^41^. *In vivo*, PLVAP expression has been correlated with disruption of the blood-brain barrier and with microvascular leakage in diabetic retinopathy^32, 42^. We confirm here that PLVAP is expressed in gut microvascularization and show for the first time that PLVAP immunoreactivity increases in digestive tissues following radiation exposure, which is known to induce vascular leakage^3^, suggesting that PLVAP may be a marker of radiation-induced microvascular damage. The number of PLVAP-positive spots in Hey2KO^endo^ mice was significantly reduced, suggesting that Hey2 deletion may protect the endothelium from radiation damage, preserving the clonogenic/stem epithelial cell compartment.

Finally, intestinal tissue exposure to high doses of ionizing radiation is known to generate late damage with a strong consequential component, i.e. the severity of chronic damage depends on the severity and duration of the acute phase^43^. In the present study, reducing the severity of the acute phase had beneficial effects on late damage, as already demonstrated with other acute therapeutic strategies, including strategies targeting epithelial protection^44^. One should, however, remain cautious regarding Hey2 inhibition. Hey2 belongs to the most prominent Notch target genes and dysregulation of Notch signaling occurs in several inherited syndromes and in cancer^45^, in the maintenance of proliferating crypt cells in the intestine^46^ and in the process of angiogenesis necessary throughout lifetime^47^. In endothelial cells, Hey2 is dependent on Notch1, but other signaling pathways are involved in Hey gene expression, and crosstalk between Notch, TGF-beta and BMP signaling has been demonstrated^37, 47^. Given that both TGF-beta and BMP may be implicated in tissue scarring and fibrosis, further studies will be necessary to investigate possible negative consequences of Hey2 deletion in different cell types, with particular emphasis on these three pathways.

In conclusion, the present work demonstrates that a strategy of gene deletion only in the endothelium is sufficient to mitigate radiation-induced proctitis in our preclinical model, confirming the cornerstone role of the endothelium in damage to healthy tissues. We also highlighted that Hey2 is involved in radiation-induced EndoMT and that Hey2 invalidation reduces EndoMT and tissue damage. The present work demonstrates for the very first time that a strategy based on the reduction of EndoMT, via Hey2 inhibition but also in principle via other molecular components of EndoMT, offers protection to radiosensitive endothelial and epithelial compartments and mitigates intestinal tissue damage. These interesting results warrant further studies of radiation-induced EndoMT and may offer new perspectives in the understanding of the radiation response of healthy tissues and obviously of tumors in the management of the effectiveness and associated side effects of radiation therapy.

## Methods

### Cell line

Primary HUVECs (C2519A pooled) were from Lonza (Verviers, Belgium) and grown at 37 °C with 5% CO_2_ in EBM-2 MV medium (Lonza).

### Cell transfection

*Hey2 expression plasmid*: 80% confluent HUVECs were transfected with pCMV6-HEY2-Myc-DDK plasmid expression vector (Clinisciences, Nanterre, France) using FuGENE (Roche Diagnostics, Meylan, France) as transfection reagent. pCMV6-AC-HA-His (Empty vector, Clinisciences) was used as control. Three days after transfection, cells were fixed for immunolabeling or treated for RNA or protein extraction. *Hey2 siRNA*: 60% confluent HUVECs were transfected with ON-TARGET plus human Hey2 siRNA and ON-TARGET control pool siRNA NT as a control, using DharmaFECT 1 as transfection reagent, as indicated by the manufacturer (GE Healthcare Dharmacon, Lafayette, CO, USA). Cells were irradiated 48 h post-transfection and harvested for mRNA extraction 7 days post-irradiation.

### Cell immunofluorescence

Seven days after 0 or 10 Gy irradiation, cells were rinsed in PBS and fixed in 4% paraformaldehyde at room temperature for 10 min. After permeabilization (0.1% Triton X100, 10 min) and blocking with 2% bovine serum albumin (1 h), primary antibodies were incubated for 1 h at room temperature: polyclonal rabbit anti-human von Willebrand factor (vWF, Dako, Les Ulis, France), monoclonal mouse anti-human alpha-smooth muscle actin, clone 1A4 (α-SMA, Dako), polyclonal goat anti-human smooth muscle 22-alpha (SM22-α), rabbit polyclonal anti-Hey2 and rabbit polyclonal anti-VE-cadherin antibodies (Abcam, Paris, France). Cells were incubated with corresponding secondary antibodies (Alexa Fluor^®^-conjugated antibodies, Life Technologies, Saint Aubin, France) for 1 h at room temperature. For double immunostaining of vWF and alpha-SMA, primary antibodies were incubated together, and after rinsing the corresponding secondary antibodies were added. Slides were mounted in Vectashield mounting medium with DAPI (Eurobio/Abcys, Courtaboeuf, France).

p120-Catenin/F-actin co-immunostaining was performed as follows: cells were rinsed, fixed, permeabilized and primary rabbit monoclonal anti-p120 catenin antibody (Abcam) was added and incubated for 1 h at room temperature. Corresponding secondary antibody (Alexa Fluor^®^-conjugated antibody, Life Technologies) was added for 1 h at room temperature. After rinsing, the CytoPainter F-actin Staining Kit – Red Fluorescence (Abcam) was used according to the manufacturer’s instructions. Slides were mounted in Vectashield mounting medium without DAPI (Eurobio/Abcys).

### Flow cytometry

Seven days after 10 Gy irradiation, control and irradiated HUVECs were washed, trypsinized and then 1 × 10^6^ cells were resuspended, fixed and permeabilized in 250 µL using BD Cytofix/Cytoperm™ Kit. After two rinses, cells were stained for 30 min at room temperature with α-SMA and vWF antibodies (Abcam). Labeled cells were centrifuged and each pellet was suspended in 500 μL of PBS containing 5% FBS. Multi-parametric analyses were performed on a BD FACSCanto™ II (3-laser, 4-2-2 configuration) for data recording and using FlowJo 7.6.5 software (FlowJo LLC). A first analysis was done on size (FSC: forward scatter)/granulometry (SSC: side scatter) parameters, to collect fixed cells and to remove fragmented cells and debris (Supplementary Fig. 2a and b). This first step allowed us to determine the gate where at least 5 × 10^4^ events per replica were recorded. Then, upon these gated events (Supplementary Fig. 2c), the vWF signal was collected on the FITC channel (filters λ_em_: 530/30 nm) after air-cooled 488 nm solid state (20-mW output) laser excitation, while the α-SMA signal was collected on the Alexa 647 channel (filters λ_em_: 660/20 nm) after air-cooled 633 nm HeNe (17-mW output) laser excitation. The specificity of the staining was previously checked by using isotype controls, sheep IgG fluorescein (Abgent, Clinisciences) and rabbit IgG, monoclonal [EPR25A] (Abcam) for vWF and α-SMA, respectively (Supplementary Fig. 3).

### Western blots

Cell total protein was extracted using RIPA buffer supplemented with phosphatase and protease inhibitors (Roche Diagnostics, Meylan, France). Protein concentration was determined using a BCA assay (Sigma Aldrich) and equal amounts of protein were resolved by SDS-PAGE. The blocked membrane was incubated with primary antibodies overnight at 4 °C. Bands were detected by using a horseradish peroxidase secondary antibody and visualized with ECL Plus reagent (GE Healthcare, Buc, France). β-Tubulin was used as loading control (Santa Cruz Biotechnology, Heidelberg, Germany).

### Quantitative real-time PCR

Total RNA was prepared with the total RNA isolation kit RNeasy Mini Kit (Qiagen, Valencia, CA). After quantification on a NanoDrop ND-1000 apparatus (NanoDrop Technologies, Rockland, DE), reverse transcription was performed with 1 µg RNA using a reverse transcription kit from Applied Biosystems (Courtaboeuf, France). Quantitative PCR was carried out on a 7900HT Fast-Real Time PCR system (Applied Biosystems) using pre-developed TaqMan® Gene Expression assays (Applied Biosystems), with GAPDH as housekeeping gene. Relative mRNA was quantified using the ΔΔCT method. DataAssist^TM^ software (Life Technologies) was used to perform hierarchical clustering with global normalization and Pearson’s correlation.

### Hey2 conditional null mice

To study the effects of *Hey2* inactivation on radiation-induced EndoMT *in vivo*, mice possessing LoxP sites on either side of exons 2 and 3 of the target gene Hey2 (Hey2^flx/flx^; Hey2^tm1Eno^ / J; The Jackson Laboratory, Bar Harbor, USA) were bred with mice with Cre recombinase expression under the endothelial promoter VE-cadherin (VECad-Cre^+/+^ mice^48^),. The following mice were used for experiments: Hey2^flx/flx^/VECad-Cre^−/−^ mice (named Hey2^flx/flx^) and Hey2^flx/flx^/VECad-Cre^+/−^ mice (named Hey2KO^endo^, see Supplementary Fig. 6).

### Mouse genotyping

Genomic tail DNA was extracted (KAPA mouse genotyping kit, Kapa Biosystem, USA) and amplified by PCR. Reverse and forward primers were provided by Life Technologies, France. Two primers were used: 5′-CTAGAGAGGACCTGGAGAGTTTAAG-3′ forward and 5′-CTGTGCCACCAGCCTTAAAACC-3′ reverse.

### Irradiation

Cell irradiation (2, 10 or 20 Gy single doses) was performed at 90% cell confluence using a ^137^Cs source (IBL 637, CisBio, Saclay, France; dose rate 1 Gy.min^−1^). For fractionated irradiation, confluent cells were exposed to 2 Gy daily fractions, 5 days a week with a weekend break. Animal irradiation procedures: the protocol (P13-18) for animal use in this work was reviewed and approved by national ethics committee no. 81, animal facilities agreement number C92-032-01. For colorectal irradiation, mice were anesthetized (1.5% isoflurane), and a single dose of 22 Gy was delivered through a 1 × 0.8 cm window centered on the colorectal region, using the Elekta Synergy^®^ Platform (Elekta S.A.S. France, Boulogne, France) delivering 4 MV X-rays at 2.6 Gy/min. For total body irradiation, mice were anesthetized with intraperitoneal injection of ketamine/xylazine and exposed to 4 MV X-rays at 2.6 Gy/min though a 30 × 30 cm window at a total dose of 12 Gy.

### Tissue harvesting, histology and immunohistology

Two and 7/8 weeks after radiation exposure, colorectal tissues were removed, fixed in 4% paraformaldehyde and embedded in paraffin. Sections (5 µm) were stained with hematoxylin-eosin-saffron for routine histological examination.

#### Radiation Injury Score (RIS)

RIS variables consisted of mucosal ulceration, epithelial atypia, thickening of the subserosa, vascular sclerosis, intestinal wall thickening, colitis cystica profunda and dystrophy of the muscularis propria (MP). Radiation injury was graded for each variable as null (0), slight (1), moderate (2) or severe (3). Dystrophy of the MP was graded as follows: 0: none; 1: dystrophy of a few muscular cell layers in contact with the submucosa; 2: dystrophy affecting less than 50% of the MP thickness; and 3: dystrophy affecting more than 50% of the MP thickness^49^.

#### Measurement of mucosal ulceration in acute samples

lengths of injured area and area affected by loss of bordering epithelium were measured using the Visiol@b^TM^2000 image analysis software (Biocom SA, Les Ulis, France), and data were expressed as percentages. The number of epithelial regeneration figures was counted on the entire damaged area (between 4 and 9 mm observable on tissue sections) and reported per mm. Multilobed glandular hyperplasia was defined as at least 2 crypt bases associated with a single crypt lumen.

#### Measurement of healthy and cuboidal epithelium in chronic samples

measurements were done on HES-stained sections, helped by specific epithelial cell staining with p120 catenin antibody using the Visiol@b^TM^2000 image analysis software (Biocom SA) on the total injured area and expressed as percentages. Healthy bordering epithelium consisted of numerous columnar epithelial cells close to each other, presenting p120 catenin-stained junctions and a basal nucleus. Cuboidal bordering epithelium consisted of cuboidal epithelial cells presenting diffuse p120 catenin staining and a centered nucleus.

Counting surviving crypts 3 days after total body irradiation: Crypts surviving irradiation were identified based on histological appearance, i.e. undilated and possessing at least 10 well-stained epithelial cells and 1 Paneth cell. Surviving crypts were counted using the Visiol@b^TM^2000 image analysis software (Biocom SA), over a total tissue length of between 1900 and 2600 µm and related to 1000 µm.

#### Immunofluorescence

longitudinal sections were deparaffinized/rehydrated, permeabilized in PBS-0.1% Triton X-100 and antigens were unmasked in citrate buffer pH = 9 (Dako). Sections were incubated with rabbit anti-alpha-sma antibody, rabbit polyclonal anti-p120 catenin or rat anti-PLVAP (Abcam) for 1 h at room temperature. Samples were then incubated with the corresponding secondary Alexa Fluor^®^-conjugated antibody (Life Technologies). For immunofluorescent double labeling: sections were incubated with rabbit polyclonal antibody to alpha-sma (Abcam) followed by the corresponding secondary Alexa Fluor^®^-conjugated antibody (Life Technologies), and incubated with sheep polyclonal antibody to vWF/FITC-conjugated antibody (Dako). Sections were mounted in Vectashield mounting medium with DAPI (Eurobio/Abcys).

*The number of PLVAP-positive spots* was quantified on 5 different fields per slide for each animal using the Visiol@b^TM^2000 image analysis software (Biocom SA). Yellow merging signals following α-SMA/vWF double fluorescence marking EndoMT were monitored by confocal analyses in a Zeiss LSM 780 confocal imaging system (Zeiss France, Marly le Roi, France).

*TUNEL staining* was performed using the *In situ* Cell Death Detection Kit (Roche Applied Science) according to the manufacturer’s instructions. After TUNEL staining, sections were incubated with rabbit monoclonal anti-p120 catenin antibody (Abcam) for 1 h at room temperature followed by the corresponding secondary antibody (Alexa Fluor^®^-conjugated antibodies, Life Technologies). Apoptotic epithelial cells were counted in about 50 crypts per tissue section and data were reported as number of TUNEL-positive cells per crypt.

### Human rectal tissues

Human tissues were obtained at the Department of Pathology, Gustave Roussy Institute (Villejuif, France) following institutional ethical guidelines and French Medical Research Council guidelines. Twenty-four patients treated for rectal adenocarcinoma with preoperative radiotherapy (45 Gy, fractions of 2 or 1.8 Gy) underwent surgical resection of tumors 5 to 7 weeks post-treatment. Specimens of normal tissue were taken adjacent to the tumor (in the irradiation field) and distant from the tumor (outside the irradiation field), making each patient his or her own control. For each patient, an RIS was applied as already described^15^. In the present study, tissue specimens from 4 patients presenting a RIS >75/100 were chosen for Hey2 immunostaining.

### Statistical analyses

Data are given as means ± SEM. Statistical analyses were performed by ANOVA, Student’s t-test or the Mann-Whitney rank sum test when appropriate, with a level of significance of p < 0.05.

## Electronic supplementary material

Supplementary Data
