# Supplementary material for: Endothelial Hey2 deletion reduces endothelial-to-mesenchymal transition and mitigates radiation proctitis in mice
Source: Sci Rep. 2017 Jul 10;7:4933. doi: 10.1038/s41598-017-05389-8 (PMC5503994; doi:10.1038/s41598-017-05389-8)
Supplement: Supplementary file 1 — Supplementary Data [file 41598_2017_5389_MOESM1_ESM.pdf]

Endothelial Hey2 deletion reduces endothelial-to-mesenchymal transition and mitigates radiation proctitis in mice.

Elodie Mintet[1,4], Jérémy Lavigne[1,4], Vincent Paget[1], Georges Tarlet[1], Valérie Buard[1], Olivier Guipaud[1], Jean-Christophe Sabourin[2], Maria-Luisa Iruela-Arispe[3], Fabien Milliat[1] and Agnès François[1*].

Supplementary data

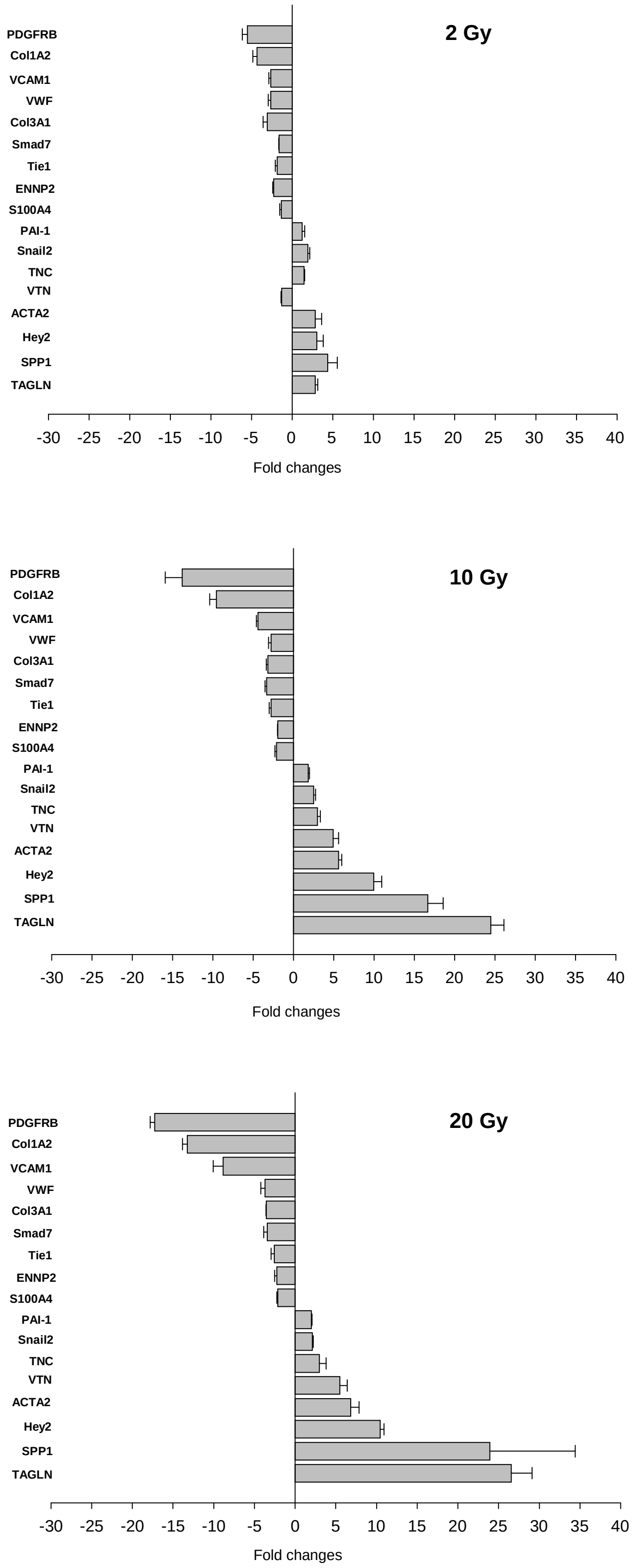

Supplementary Figure 1 – Irradiation induces phenotypic conversion of endothelial cells resembling EndoMT. Fold changes in mRNA expression profiles obtained in irradiated HUVECs 7 days after 2, 10 or 20 Gy exposure. Data are representative of 3 independent experiments performed in triplicate. Only statistically significant fold changes versus 0 Gy are shown ($p<0.05$).

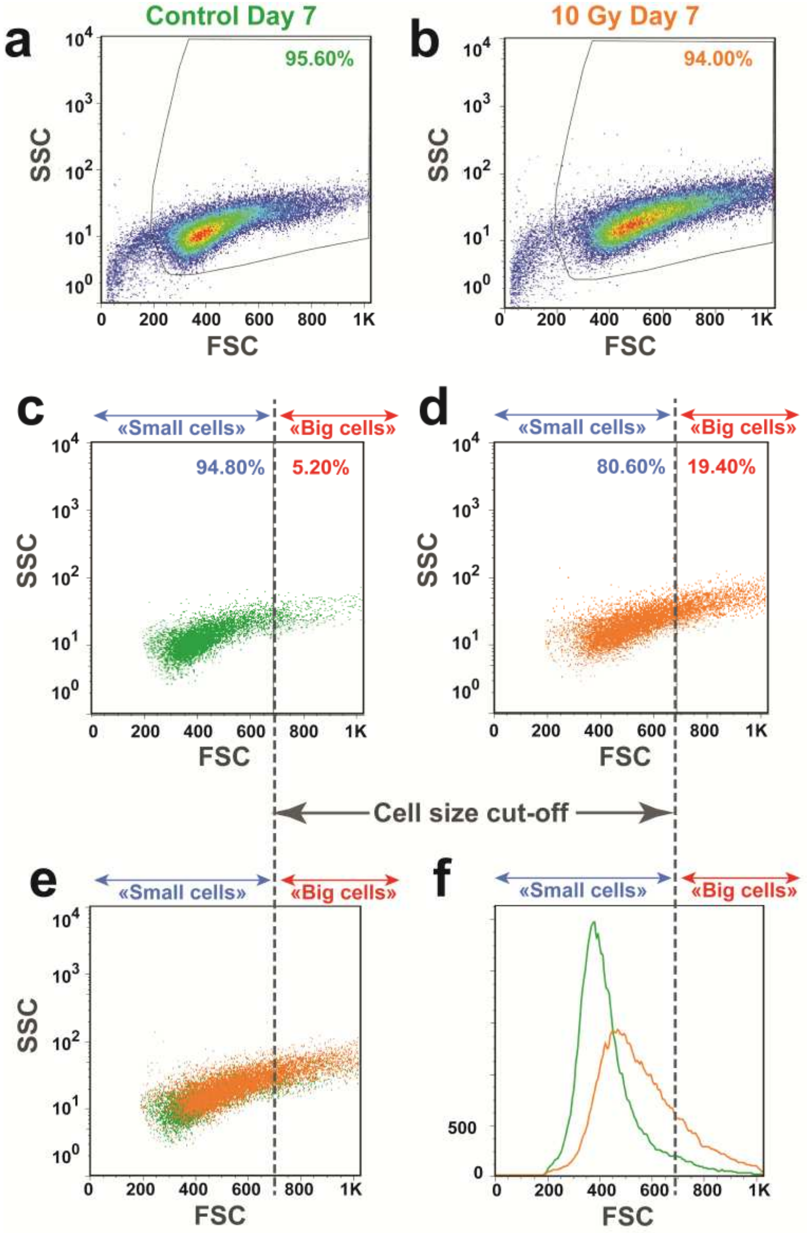

Supplementary figure 2 – Gating and cell size cut-off based on FSC measurements. Gating on living cell population is represented for control day 7 on panel (a) and on panel (b) for 10 Gy day 7. Cell size cut-off was set at around 5% for big cells on control day 7 (c) while this population represents around 19% on 10 Gy day 7 (d). Overlap feature and cell size cut-off of the two conditions are depicted on panels (e) and (f), as dot plot and histogram representation, respectively. Data represent one representative experiment among three independent experiments.

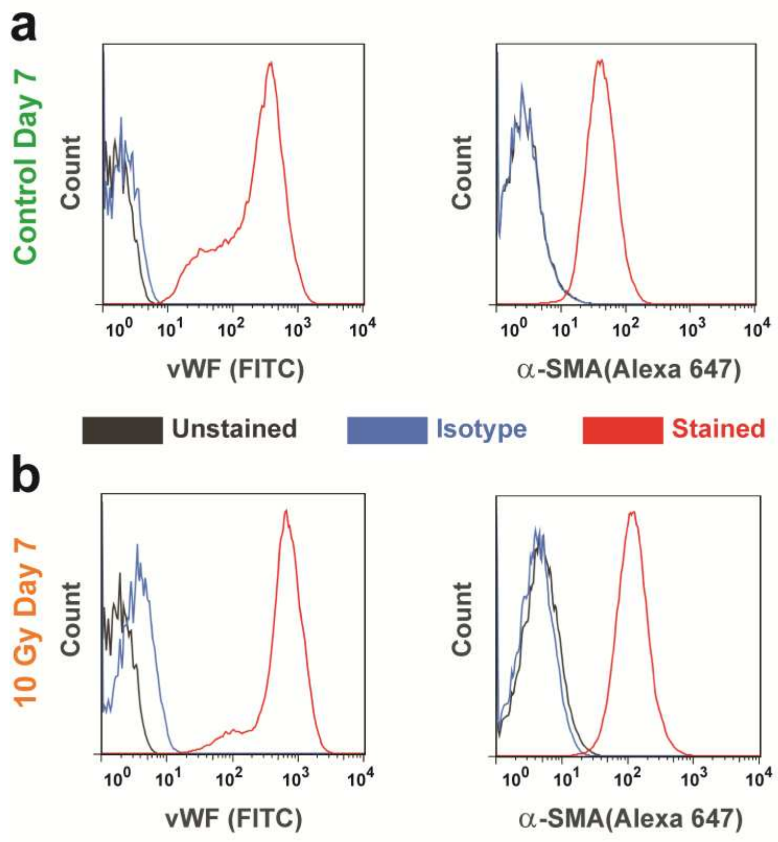

Supplementary figure 3 – Isotype controls for α-SMA/VWF double-staining on HUVECs. Isotype antibodies (blue curves) were compared with unstained (gray curves) and stained cells (red curves) for control day 7 (a) and 10 Gy day 7 (b). Data represent one representative experiment among three independent experiments. For unstained and both isotypes, no overlap or false-positive signal was observed in the corresponding stained samples.

a

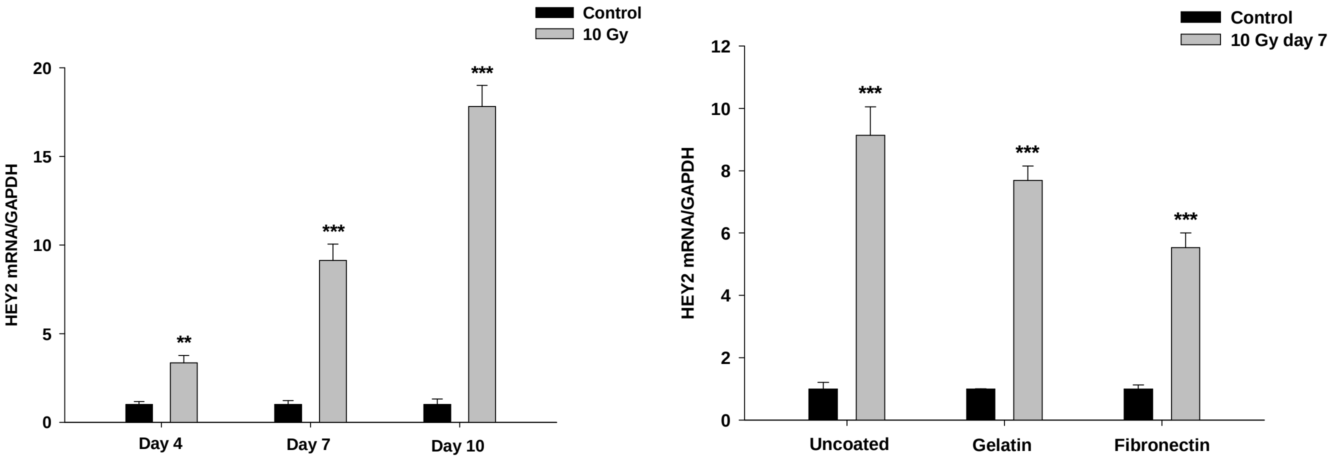

b

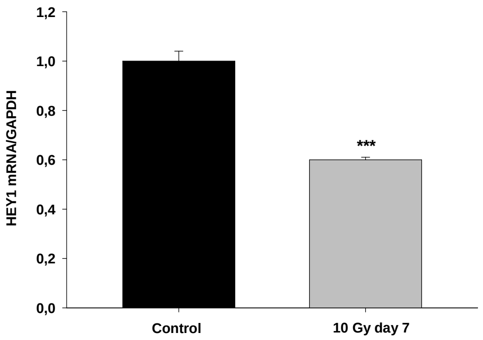

Supplementary figure 4 – Radiation-induced Hey2 overexpression is progressive with time and independent of plastic coating and occurs together with Hey1 downregulation. (**a**) Hey2 mRNA expression in 10 Gy-irradiated HUVECs was measured by qPCR 4, 7 and 10 days post-exposure. For the 10 Gy 7 days time point, Hey2 mRNA expression was measured in HUVECs cultured on uncoated, gelatin- or fibronectin-coated plastic with no influence on Hey2 expression modifications. (**b**) Hey1 mRNA expression levels in control and 10 Gy-irradiated HUVECs. Data are representative of three independent experiments performed in triplicate. **$p<0.01$; ***$p<0.001$.

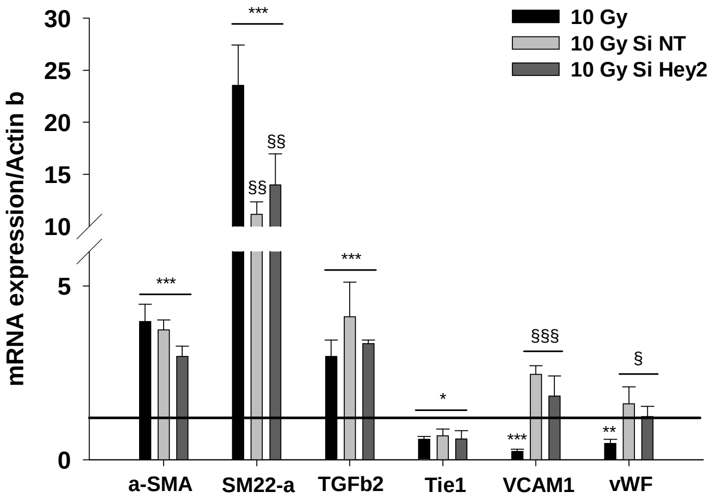

Supplementary figure 5 – mRNA expression levels measured by qPCR in 10 Gy-irradiated, previously untransfected, non-targeting (NT) siRNA- or Hey2-SiRNA-transfected HUVECs compared with control unirradiated cells (retrieved to 1, barr), 7 days after irradiation. Data are the mean ± SD of one experiment performed in triplicate. *p<0.05; **p<0.01; ***p<0.001 compared with unirradiated cells; § p<0.05; §§ p<0.01; §§§ p<0.001 compared with 10 Gy untransfected cells.

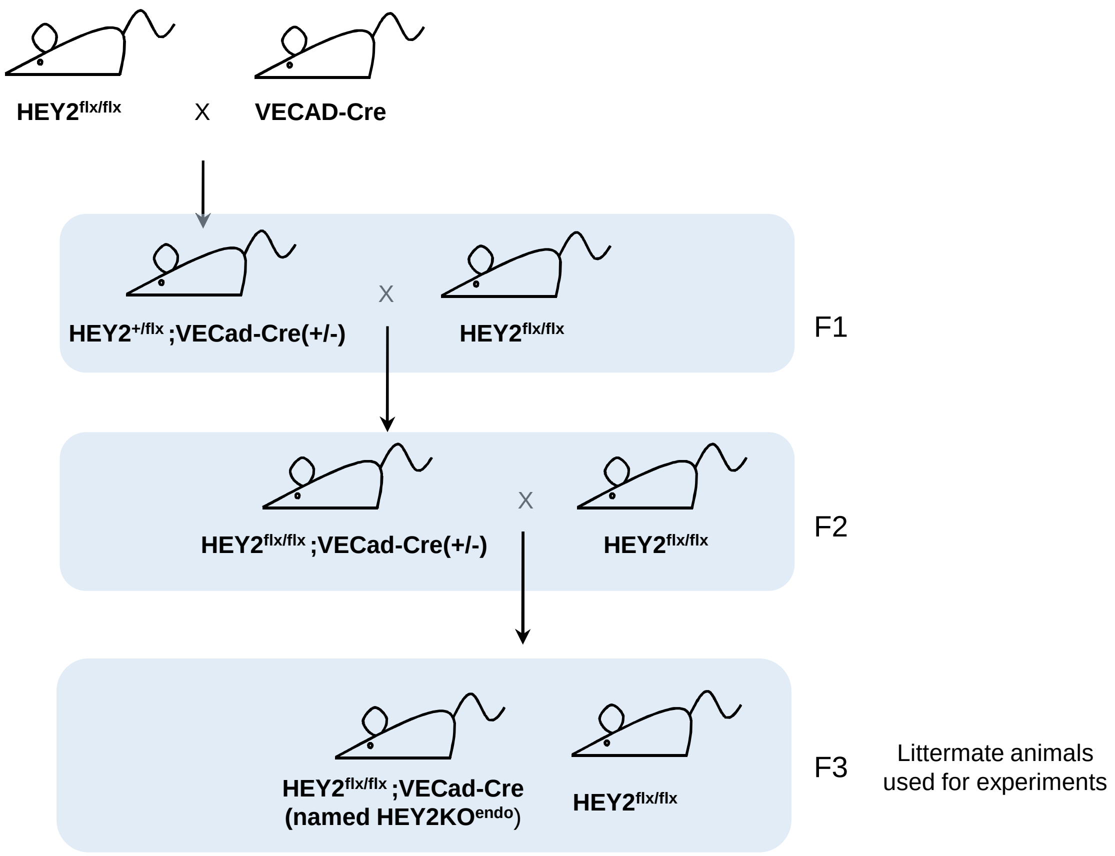

Supplementary figure 6 – Global breeding scheme for producing transgenic mice for experiments.
